# The Role of Pharmaceutical Innovation in Clinical Practice Guidelines for Chronic Diseases

**DOI:** 10.1155/2024/5877687

**Published:** 2024-03-12

**Authors:** Meaghan Roach, Natalie Land, Jennifer Hernandez, Reina Rau, Jacquelyn W. Chou, Stacey S. Hickson, Danielle F. Rollmann, J. Ross Maclean

**Affiliations:** ^1^PRECISIONheor, 2 Bethesda Metro Center, Suite 850, Bethesda, MD 20814, USA; ^2^Janssen Pharmaceuticals Inc., 700 US Highway 202, Raritan, NJ 08869, USA

## Abstract

**Background:**

Over the last 25 years, clinical practice guidelines have emerged as a means to standardize and improve care. As pharmaceutical innovations develop, guidelines are updated to incorporate new interventions. However, the extent to which pharmacotherapies are represented as treatment options in guideline recommendations has not been well elucidated. This study aimed to quantify the role pharmacotherapy has played in clinical practice guidelines across a range of chronic diseases over the past 20 years.

**Methods:**

Clinical practice guidelines published from 2000 to 2021 were identified for five chronic diseases: ischemic heart disease (IHD), non-small cell lung cancer (NSCLC), chronic obstructive pulmonary disease (COPD), Alzheimer's disease (AD), and type 2 diabetes (T2D). Guidelines were reviewed and data on treatment recommendations were collected, including the type of intervention, line of therapy, and, for pharmacotherapies, year of regulatory approval and year of inclusion in guidelines.

**Results:**

In total, 92 clinical practice guidelines were reviewed. Among the 184 discrete recommended interventions across the five disease areas, 146 (79.3%) were pharmacotherapies, 21 (11.4%) were behavioral modifications, 6 (3.3%) were surgical interventions, and 11 (6%) were other interventions. Across guidelines, when a line of therapy was specified, behavioral modifications and pharmacotherapies were most frequently recommended as first-line interventions, whereas surgical interventions were more often recommended for subsequent lines of treatment. The time from regulatory approval of novel pharmacotherapies to inclusion in guideline recommendations varied considerably by disease area and geography.

**Conclusions:**

Across the reviewed disease areas, behavioral interventions and pharmacotherapies are shown to be critical components of clinical practice. Over the last 20 years, novel pharmaceutical innovations have been incorporated into clinical practice guideline recommendations; however, with varying speeds of adoption. Given the increasing pace of pharmacologic innovation, timely updates of clinical practice guidelines are critical to evolving the standard of care and practicing evidence-based medicine.

## 1. Introduction

As modern medicine has rapidly progressed, deaths from communicable diseases have begun to decrease. However, countries across the world, including low-, middle-, and high-income countries, are experiencing a rising burden of noncommunicable chronic diseases. In high-income countries specifically, noncommunicable chronic diseases account for nine of the ten leading causes of death [1]. Pharmaceutical products have long been believed to be the mainstay of effective treatment for chronic diseases. Indeed, emphasis has been placed on pharmacologic treatments for many common chronic diseases by both the World Health Organization (WHO) and the General Assembly of the United Nations [2, 3]. However, the extent to which pharmacotherapies represent treatment recommendations and primary treatment recommendations across chronic diseases has not been well elucidated.

Clinical practice guidelines from professional societies and organizations have emerged over the last 25 years as a means to improve delivery in what is often a rapidly changing treatment landscape [4–6]. Guidelines are intended to summarize the best and most current prevailing evidence on available treatments across all modalities and to recommend the appropriate use of each treatment based on the evidence. Clinical practice guidelines provide practitioners with a consolidated, critical review of the growing body of evidence for treatment of conditions, intended to improve the quality of patient care [7]. They provide an opportunity to observe and objectively measure the relative importance of different types of interventions in patient care and in improving clinical outcomes over time, potentially reducing variation in care delivery.

As pharmaceutical innovation has occurred rapidly over the past decades, clinical practice guidelines across a broad range of conditions have been modified to incorporate new therapies into their recommendations. This study aimed to assess, based on clinical practice guidelines, the role pharmacotherapy and other treatment options have played in treatment across a range of high burden chronic diseases over the past 20 years. Specifically, existing clinical practice guidelines across a range of chronic diseases were evaluated to determine the distribution of the types of interventions recommended for each disease area, including by line of therapy, and the pace of guideline adoption of pharmacotherapy innovations.

## 2. Methods

### 2.1. Disease Selection

In order to capture clinical practice guidelines representative of a broad range of chronic diseases that have substantially impacted society, five diseases were selected for review from the WHO top ten causes of mortality in high-income countries during the year 2000 [1]. To ensure diversity among the disease areas reviewed, the top ten causes of death were first categorized by disease area, and the highest-ranked indication from each disease area was selected. For the indications with several subtypes, the subtype that contributed the highest number of deaths was selected. The resulting five diseases included ischemic heart disease (IHD), non-small cell lung cancer (NSCLC), chronic obstructive pulmonary disease (COPD), Alzheimer's disease (AD), and type 2 diabetes (T2D) (Table 1).

### 2.2. Guideline Identification and Data Abstraction

A targeted literature search was conducted to identify clinical practice guidelines published by relevant United States (US), European (EU), and global healthcare organizations for each of the five selected diseases during the years 2000 to 2021. Identified guidelines that were available in the public domain and were readily accessible online were included. All identified guidelines were reviewed, and data on treatment recommendations were abstracted when the guidelines issued a positive recommendation for the use of a given intervention. Interventions were categorized by recommended line of therapy when specified. Interventions recommended for specific subpopulations (e.g., elderly, pediatric, and pregnant populations), management of comorbidities, or prevention of the disease were not included. For example, interventions recommended in T2D guidelines for blood pressure, lipid, and cardiovascular disease management were not included. If an intervention was not mentioned, not recommended, or if the strength of the recommendation was weak as defined by the respective guideline, no data were abstracted (Supplemental Table S1). If the guideline did not provide a strength of recommendation, statements in the guideline were used to discern whether the guideline was recommending a given therapy. Recommendations for all types of treatments such as pharmacotherapies, surgery, behavioral or lifestyle modifications, and others were included. Data abstraction was performed by three independent researchers (R1, R2, and R3) with master's level training. A senior researcher (R4) reviewed the final data and settled any discrepancies.

### 2.3. Analysis

For each disease, the total number of discrete interventions within and across guidelines were categorized and counted by type of treatment to determine the proportion of recommended interventions that were pharmacotherapies, surgical interventions, behavioral or lifestyle modifications, or other. Some guidelines consistently specified the recommended line of therapy for an intervention while others did not. For recommendations that explicitly specified a line of therapy, the findings were further stratified by line of therapy to determine the distribution of types of treatments that were recommended for first-line versus subsequent lines. If guidelines did not explicitly include a line of therapy with a recommendation, the intervention was categorized as “not recommended by line.” Additionally, a temporal analysis was performed to assess how recommendations for interventions changed over time and when new interventions were incorporated into guidelines. For pharmacotherapies, data on the year of approval by the Food and Drug Administration (FDA) and by the European Medicines Agency (EMA) was collected to assess the timing of inclusion in the guidelines relative to regulatory approval dates [11, 12].

Specific methodological considerations were required for some disease areas due to unique circumstances and variability in clinical practice guidelines and recommendations. For example, recommendations within and across disease areas varied in whether they specified a line of therapy for the intervention; some guidelines made recommendations for a broad class of drugs rather than specifying recommendations at the individual drug level; and the frequency of published guideline updates varied widely across diseases. If a guideline made recommendations at the drug class level, the class as a whole was counted as one discrete intervention. These considerations are detailed below for each respective disease.

#### 2.3.1. IHD

While EU IHD guidelines consistently specified the line of treatment across recommendations, US guidelines were less consistent and only specified the line of treatment in about half of the recommendations. A full US guideline update has not been issued since 2012, and therefore, no new pharmacotherapies have been included in the US guidelines since then. The 2016 US and 2017 EU guidelines were partial updates specific to dual antiplatelet therapy (DAPT) that did not provide recommendations on other therapies.

#### 2.3.2. NSCLC

Early NSCLC guidelines made recommendations primarily at the treatment class level; however, over time, recommendations became more specific and were made at the drug level. While EU guidelines published by the European Society for Medical Oncology (ESMO) included other forms of treatment beyond pharmacotherapies such as surgery and radiotherapy, not all American Society of Clinical Oncology (ASCO) guidelines included recommendations for these other types of interventions. Beginning in 2009, ASCO guidelines limited their scope to recommendations for chemotherapy and biologic therapy for stage IV NSCLC due to the large volume of literature the ASCO guideline committee would have to review [13]. The 2011 ASCO guideline was a focused update, providing recommendations only on maintenance chemotherapy. Additionally, the 2020 and 2021 ASCO guidelines were partial updates where the former focused on treatments for patients without driver alterations and the latter on patients with driver alterations. Guidelines from the National Comprehensive Cancer Network (NCCN) were not included due to the lack of public availability for past years' NSCLC guidelines.

#### 2.3.3. COPD

Recommendations for pharmacotherapies were made primarily at the class level rather than at the drug level. Across all guidelines, no recommendations were made with specifications for the line of treatment; thus, the analysis of proportions of types of interventions recommended for the first versus subsequent lines was not possible.

#### 2.3.4. AD

No treatments for AD itself existed until the recent approval of aducanumab in 2021; thus, data were instead abstracted for recommendations regarding treatments for the management of cognitive symptoms in AD patients rather than treatment of the underlying disease. The 2015 European Federation of Neurological Sciences/European Academy of Neurology guideline was a focused update only providing recommendations for combination therapy and not including recommendations for other therapies.

#### 2.3.5. T2D

Guidelines for the treatment of T2D primarily made recommendations for pharmacotherapies at the class level instead of by specific drug; thus, analyses for this disease area were performed at the class level.

## 3. Results

In total, 92 clinical practice guidelines from 2000 to 2021 were reviewed, representing five of the top ten most common causes of mortality among high-income countries [1]. Supplementary Table S2 provides a summary of all guidelines that were abstracted and included in this review. Across the five disease areas, there were more US guidelines issued than EU or global guidelines (US: 47; EU: 24; Global: 21), with considerable nuance within each disease area (Table 2). T2D, with a total of 36 guidelines (29 US; 5 EU; 2 Global), and COPD with a total of 23 guidelines (4 US; 0 EU; 19 global), had the greatest number of guidelines as these disease areas had organizations that published updates annually or almost annually within our selected timeframe (e.g., 2000 to 2021). NSCLC guidelines were also published fairly frequently with a total of 19 guidelines were readily available (7 US; 12 EU; 0 Global). AD and IHD guidelines were published very infrequently with only a total of 7 guidelines for AD (4 US; 3 EU; 0 Global) and 6 for IHD (3 US; 3 EU; 0 Global).

Guidelines covered recommendations across behavioral modifications, pharmacological treatments, and surgical interventions. Among the 184 discrete recommended interventions across the five disease areas, 146 (79.3%) were individual pharmacotherapies or classes of pharmacotherapies, 21 (11.4%) were behavioral/lifestyle modifications, 6 (3.3%) were surgical interventions, and 11 (6%) were other interventions (Table 3).

### 3.1. Interventions by Line of Therapy

Across guidelines, only behavioral and lifestyle modifications and pharmacotherapies were recommended as first-line therapies. Of the six surgical interventions represented across the five disease areas, only those for IHD (EU only) were specified by line of therapy, none of which were recommended for first-line treatment. When included, behavioral or lifestyle interventions were consistently recommended as initial or first-line treatment, often in conjunction with pharmacotherapies. For example, US, EU, and global guidelines for T2D consistently recommended behavioral and lifestyle interventions (e.g., diet and physical activity) along with pharmacotherapy (e.g., metformin) as first-line treatments to manage blood glucose levels. Overall, of the total 21 recommended behavioral modifications across all five disease areas, 18 were specified by line of therapy, and 100% of those were recommended as first-line therapy.

**Figure 1:**
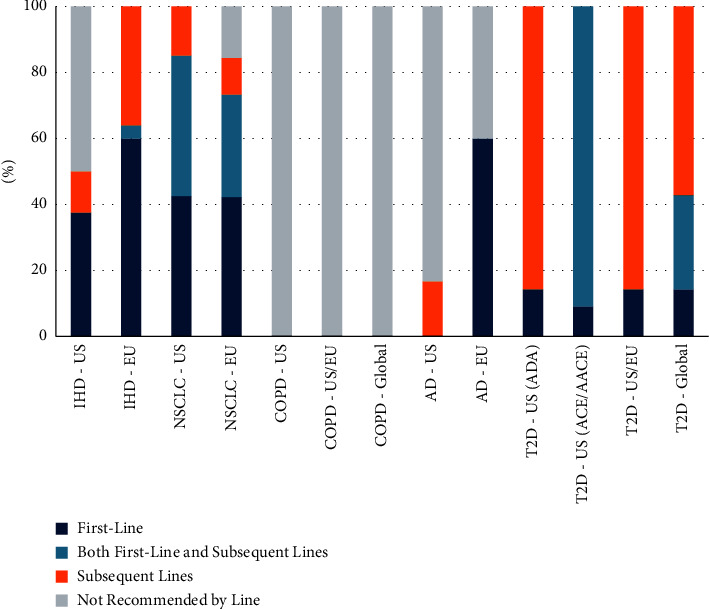
Proportion of pharmacotherapies recommended by the line of treatment. ^*∗*^Data represents the line of therapy a pharmacotherapy was ever recommended for. For example, if a pharmacotherapy was first recommended for first-line and was then later recommended for subsequent lines, it is counted as recommended for both first and subsequent lines. IHD = ischemic heart disease; NSCLC = non-small cell lung cancer; COPD = chronic obstructive pulmonary disease; AD = Alzheimer's disease; T2D = type 2 diabetes; ADA = American diabetes association; ACE = American college of endocrinology; AACE = American association of clinical endocrinology; US = United States; and EU = European Union.

Pharmacotherapy recommendations were more nuanced, as recommendations were made at both the class level and the product level depending on the disease area and organization, and the line of therapy was not always specified (Figure 1). Specifically, all T2D guidelines and most NSCLC and IHD guidelines, included the line of therapy recommendations. However, no treatment recommendations were specified by the line of therapy for COPD guidelines, and only some were specified by the line of therapy for AD guidelines. Overall, 29.5% of pharmacotherapy recommendations were for first-line treatment, 20.7% were either first or subsequent lines, and 17.6% were for subsequent lines. The remaining 32.2% were not specified as a line of therapy.

One of the disease areas that consistently recommended pharmacotherapies by line of treatment was NSCLC, among which the majority were recommended for first-line treatment. In EU (ESMO) guidelines for NSCLC, the line of treatment for recommended pharmacotherapies was specified 84.4% of the time, among which 50% (19/38) were recommended for first-line, 13.2% (5/38) were recommended for subsequent lines, and 36.8% (14/38) were recommended for both first-line and subsequent lines of therapy. In US (ASCO) guidelines, a line of treatment was specified for all recommended pharmacotherapies, among which 42.6% (20/47) were recommended for first-line, 14.9% (7/47) were recommended for subsequent lines, and 42.6% (20/47) were recommended for both first-line and subsequent lines of therapy.

Six surgical interventions were recommended in the reviewed guidelines. Only those for IHD (EU only) were specified by line of therapy, all of which were specified for subsequent lines of treatment including the second line and beyond. Across the disease areas, only four surgical interventions were newly recommended during the study review period. All four of these were for the treatment of NSCLC, and the line of therapy was not specified.

### 3.2. Pace of Guideline Adoption of Pharmacotherapy Innovations

**Figure 2:**
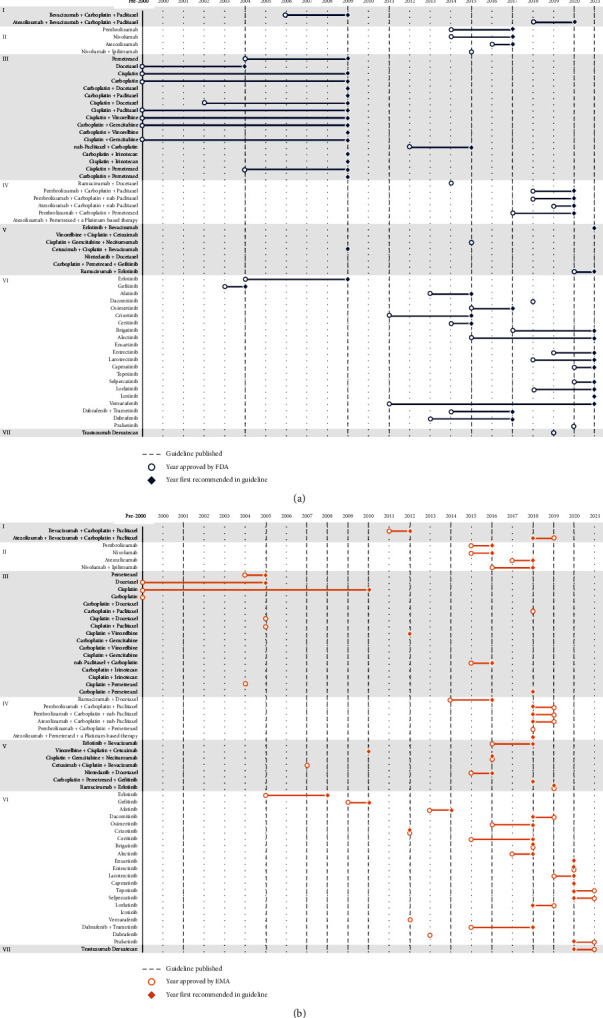
Pace of pharmacotherapy adoption in NSCLC (a) US (ASCO) and (b) EU (ESMO) guidelines. Classes of pharmacotherapies: (I) antiangiogenic + chemotherapy/+immunotherapy; (II) Checkpoint inhibitors; (III) chemotherapy; (IV) immunotherapy + chemotherapy; (V) tyrosine kinase inhibitor combination therapy; (VI) tyrosine kinase inhibitors; (VII) antibody drug conjugate. *Note*. Carboplatin and cisplatin were approved in Europe prior to establishment of EMA. NSCLC = non-small cell lung cancer; ASCO = American society of clinical oncology; ESMO = European society for medical oncology; US = United States; EU = European Union; EMA = European medicines agency.

As pharmaceutical innovations emerged along with supportive clinical evidence during the guideline review period, new pharmacotherapies were adopted into guidelines. However, how quickly guideline recommendations were updated to include these new therapies varied across disease areas and geography. For example, EU guidelines for IHD have included recommendations for new pharmacotherapies which have yet to be incorporated into US guidelines. Of note, no new therapies have been included in US guidelines for IHD since 2012, as that is the last time the US issued a full guideline update. In addition, delays in adoption of new therapies can be observed in NSCLC. Among the 31 pharmacotherapies for NSCLC that were approved since 2000 in the US, 12 were recommended in the subsequent US guideline (ASCO), while incorporation into recommendations for 16 of the new pharmacotherapies was delayed by one guideline, and 3 were delayed by two or more guidelines. In the EU NSCLC guidelines (ESMO), fewer delays were observed. Among the 41 pharmacotherapies that were approved since 2000 in the EU, 35 were recommended in the subsequent ESMO guidelines, 5 were delayed by one guideline, and 1 was delayed by two guidelines (Figure 2).The pace of adoption figures for the other four diseases are presented in the Supplementary Materials. Comparison of the pace of adoption across disease areas from time of approval to inclusion in the guidelines was not pursued due to variability in the timing of organizations' guideline releases for reasons unrelated to the introduction of innovation.

## 4. Discussion

Published clinical practice guidelines provide an opportunity to objectively assess the relative importance of different intervention options and the extent to which and speed at which novel treatments are embraced. This review sought to evaluate how pharmacotherapies, behavioral/lifestyle modifications, and surgeries or other interventions were represented in clinical practice guidelines across high-burden chronic diseases. Past work in this field has sought to standardize methodology for developing clinical practice guidelines, including how to best review, report, publish, and update guidelines [14, 15]. However, to the authors' knowledge, no review to date has sought to assess the role therapeutic options (e.g., behavioral/lifestyle modifications, pharmacotherapies, surgical interventions) have played in treatment across a range of chronic diseases. In doing so, it was found that lifestyle and behavioral modifications along with pharmacotherapies consistently dominated initial treatment recommendations. Pharmacotherapy represented approximately 79% of recommended interventions in clinical practice guidelines over the past 20 years across the five disease areas analyzed.

However, substantial nuance was also found both across and within disease areas. While some clinical practice guidelines provided intervention recommendations for specific pharmacotherapy, others recommended pharmacotherapies only on a class level. Additionally, some guidelines included recommended line of treatment while others did not. Guidelines for T2D consistently recommended interventions by line of treatment, providing a clear process for clinicians to follow. Conversely, COPD guidelines did not recommend any interventions by line of treatment. One such reason may be that 79% (19/24) of the COPD guidelines were global in nature, necessitating that the guidelines had to be general enough to be relevant across countries with differing healthcare systems and drug approval processes.

Clinical practice guidelines for some of the disease areas that were reviewed (e.g., IHD, T2D) incorporated behavioral/lifestyle modifications as core recommendations. In these guidelines, behavioral/lifestyle interventions were reliably recommended as initial or first-line treatments. True optimization of patient care also includes prevention of these and other diseases through behavioral interventions [16]. While preventive guidelines were not included in this review, it is imperative to highlight the importance of behavioral and lifestyle modifications for prevention of these and other diseases. As such, preventive guidelines may be more likely to focus on behavioral and lifestyle modifications, so exclusively looking at treatment guidelines may produce a limited capture of the overall impact and involvement of behavioral interventions. Despite not incorporating preventive guidelines in this review, our findings highlight the importance of behavioral and lifestyle modifications in managing the burden of disease.

Though not the focus of this review, it is important to recognize the complex nature of treatment recommendations and decision-making processes that occur when a patient is first diagnosed. While clinical practice guidelines are developed to provide guidance for clinicians making treatment recommendations for patients, multiple other factors–including clinician and patient preferences, patient access to care, and clinician experience–factor into the decision-making process. As pharmaceutical innovation continues and the number of treatment options increases across diseases, integrating them appropriately into guidelines along with prevention and other treatment options, such as surgery, will be important.

Substantial variability was observed in the frequency of clinical practice guideline publication. Some disease areas and representative bodies produced guidelines annually, while other guidelines were updated less frequently, leading to the existence of novel therapies that have yet to be incorporated in guideline recommendations. For example, the latest US IHD guideline identified was published in 2016, but was a limited update to the 2012 guideline. However, several new pharmaceutical treatments (e.g., apixaban, edoxaban, and ivabradine) have been approved for use by the FDA since 2012 and thus have yet to be included in the IHD guideline recommendations. More consistent guideline updates across disease areas could better equip practitioners with information on the latest innovations and impact patients' experiences and treatment journeys. Further, due to the variability in the frequency of new guideline releases, measuring the time to guideline adoption of a new pharmacotherapy may not be reflective of the actual pace of adoption of a new pharmacotherapy in clinical practice. Further research may advance our understanding of the rate of adoption of innovation into guidelines. Specifically, studies using real-world data to identify the lag in time between when treatments begin to appear in claims data relative to regulatory approval and primary data collection from clinicians in routine practice settings related to the emerging science leading guideline updates.

### 4.1. Strengths and Limitations

This review increases our understanding of the important role that pharmacotherapies, behavioral modifications, and other types of interventions play in treating some of the most common chronic conditions and top causes of mortality. The present study sought to include a representative range of the top causes of mortality in high-income countries by including conditions across cardiology, oncology, pulmonology, neurology, and endocrinology. However, this review represents a nonrandom sample of guidelines that have been developed over the past two decades. While our disease selection approach was systematic and aimed for a diverse distribution of disease areas, three of these highest mortality diseases were internal medicine diseases where surgery is presently not the dominant treatment option. While innovative medicines continue to change the treatment paradigm, we recognize that surgical treatment may be the most appropriate or only care for a patient across innumerable life-threatening and acute conditions.

Additionally, this study was limited to guidelines that were publicly available. Specifically in the case of NSCLC, the NCCN guidelines, which are recognized as prominent clinical practice guidelines in oncology, were not included in our analyses as the majority of these guidelines were not publicly available. In addition, we recognize the difference between guidelines (as studied in this project) and pathways. The latter may be local adaptations of the more “formal” academic guidelines to reflect practices and preferences at a specific health system or institution, but are often proprietary and confidential, thus beyond the scope of this study.

## 5. Conclusion

This study sought to provide a summary of the treatment recommendations found in clinical practice guidelines representative of a range of high burden chronic diseases to better understand the importance of pharmacotherapy. In doing so, it was observed that first-line treatment recommendations are primarily comprised of lifestyle and health behavior modifications, closely followed by pharmaceuticals. Pharmacotherapy represents approximately 79% of all recommended interventions in clinical practice guidelines across IHD, NSCLC, COPD, T2D, and AD over the past 20 years. Guideline adoption of new drugs is a function of the benefit to risk profile, though the speed of guideline adoption varies by disease and geography. Given the pace of pharmacologic innovation, timely updates of clinical practice guidelines are critical to evolving the standard of care and practicing evidence-based medicine.

## Figures and Tables

**Table 1:** Disease indications included.

WHO top 10 causes of death 2000 (high-income^a^ countries)			Specific area/subindication reviewed	Rationale for inclusion/exclusion
Rank	Indication	Disease area		
**1**	**IHD**	**Cardiovascular**	**Stable IHD**	There were 360,900 deaths reported from IHD in 2019: 104,280 from acute myocardial infarction; 4,379 from other acute IHD; 229,494 from atherosclerotic cardiovascular disease or atherosclerotic heart disease [8]. It was assumed that the ICD codes that most nearly match stable IHD are those for from atherosclerotic cardiovascular disease or atherosclerotic heart disease
2	Stroke	Cardiovascular	—	A cardiovascular indication (IHD) was already selected for inclusion as it was ranked higher
**3**	**Trachea, bronchus, lung cancer**	**Oncology**	**Advanced/distant NSCLC**	NSCLC is the most common type of lung cancer [9, 10], and the most advanced stage of NSCLC was selected as it is associated with the poorest survival
**4**	**COPD**	**Pulmonary/respiratory**	**Management of COPD exacerbations**	There were 119,291 deaths in 2019 from COPD, unspecified—far more than any other specific indication (bronchitis, not specified as acute or chronic; unspecified chronic bronchitis; emphysema) [8]
5	Lower respiratory infections	Pulmonary/respiratory	—	A pulmonary/respiratory indication (COPD) was already selected for inclusion as it was ranked higher
6	Colon and rectum cancers	Oncology	—	An oncology indication (NSCLC) was already selected for inclusion as it was ranked higher
**7**	**AD and other dementias**	**Neurology**	**Management of cognitive symptoms of AD**	Alzheimer's disease was selected over other dementias as it was responsible for 121,499 deaths overall in 2019 whereas unspecified dementia was responsible for 97,484 [8]
**8**	**Diabetes mellitus**	**Endocrinology**	**T2D**	Type 1 (insulin-dependent) diabetes was responsible for 2,703 deaths in 2019. Type 2 (noninsulin dependent) diabetes was responsible for 21,879 deaths in 2019 [8]. Type2 Diabetes was chosen as it contributed higher mortality
9	Breast cancer	Oncology	—	An oncology indication (NSCLC) was already selected for inclusion as it was ranked higher
10	Stomach cancer	Oncology	—	An oncology indication (NSCLC) was already selected for inclusion as it was ranked higher

^a^Selection based on world bank list of high-income economies. Bold out rows indicate disease areas that were not included in this review. WHO = world health organization; IHD = ischemic heart disease; NSCLC = non-small cell lung cancer; COPD = chronic obstructive pulmonary disorder; AD = Alzheimer's disease; T2D = type 2 diabetes; ICD = international classification of diseases.

**Table 2:** Number of guidelines reviewed by geographic region.

Disease area	US guidelines	EU guidelines	Global guidelines	Total
IHD	3	3	0	6
NSCLC	7	12	0	19
COPD	4	1	19	24
AD	4	3	0	7
T2D	29	5	2	36
Total	47	24	21	92

IHD = ischemic heart disease; NSCLC = non-small cell lung cancer; COPD = chronic obstructive pulmonary disease; AD = Alzheimer's disease; T2D = type 2 diabetes.

**Table 3:** Recommended interventions by disease area.

Disease area	IHD	NSCLC	COPD	AD	T2D	Total
Pharmacotherapies	28	61	40	6	11	146
Behavioral/lifestyle modifications	10	1	2	0	8	21
Surgical interventions	2	4	0	0	0	6
Other interventions	2	3	5	1	0	11
Total discrete interventions	42	69	47	7	19	184

IHD = ischemic heart disease; NSCLC = non-small cell lung cancer; COPD = chronic obstructive pulmonary disease; AD = Alzheimer's disease; T2D = type 2 diabetes.

## Data Availability

The data underlying this article are available from sources in the public domain: American Association of Clinical Endocrinology (AACE), American Academy of Family Physicians (AAFP), American Association for Thoracic Surgery (AATS), American College of Cardiology (ACC), American College of Cardiology Foundation (ACCF), American College of Endocrinology (ACE), American College of Physicians (ACP), American Diabetes Association (ADA), American Heart Association (AHA), American Psychiatric Association (APA), American Society of Clinical Oncology (ASCO), American Thoracic Society (ATS), Department of Defense (DoD), European Association for the Study of Diabetes (EASD), European Academy of Neurology (EAN), European Federation of Neurological Sciences (EFNS), European Respiratory Society (ERS), European Society of Cardiology (ESC), European Society for Medical Oncology (ESMO), Global Initiative for Chronic Obstructive Lung Disease (GOLD), International Diabetes Federation (IDF), Ontario Health (OH), Preventive Cardiovascular Nurses Association (PCNA), Society for Cardiovascular Angiography and Interventions (SCAI), Society of Thoracic Surgeons (STS), and Department of Veteran Affairs (VA).

## Supplementary Materials

Supplementary Materials Supplementary Table S1: Guideline's Strength of Recommendations Definition. Supplementary Table S2: Guidelines Abstracted by Disease Area. Supplementary Figure S3: Pace of Pharmacotherapy Adoption in IHD A) US and B) EU Guidelines. Supplementary Figure S4: Pace of Pharmacotherapy Adoption in COPD A) US Guidelines. Supplementary Figure S5: Pace of Pharmacotherapy Adoption in AD A) US and B) EU guidelines. Supplementary Figure S6: Pace of Pharmacotherapy Adoption in T2D A) US (ADA), B) US (AACE/ACE), and C) EU Guidelines.
