# Supplementary material for: The Role of Pharmaceutical Innovation in Clinical Practice Guidelines for Chronic Diseases
Source: Int J Clin Pract. 2024 Mar 12;2024:5877687. doi: 10.1155/2024/5877687 (PMC10950408; doi:10.1155/2024/5877687)
Supplement: Supplementary Materials — Supplementary Table S1: Guideline's Strength of Recommendations Definition. Supplementary Table S2: Guidelines Abstracted by Disease Area. Supplementary Figure S3: Pace of Pharmacotherapy Adoption in IHD A) US and B) EU Guidelines. Supplementary Figure S4: Pace of Pharmacotherapy Adoption in COPD A) US Guidelines. Supplementary Figure S5: Pace of Pharmacotherapy Adoption in AD A) US and B) EU guidelines. Supplementary Figure S6: Pace of Pharmacotherapy Adoption in T2D A) US (ADA), B) US (AACE/ACE), and C) EU Guidelines. [file 5877687.f1.docx]

Supplementary Material

**Supplementary Table S1. Guideline’s Strength of Recommendations Definition**

| Guideline (Year) | How Strength of Recommendation was Defined | | Excluded Recommendations Based on Recommendation Grade^ | |
| --- | --- | --- | --- | --- |
| *IHD* | | | | |
| US*  (2012, 2014, 2016) | **Class I** = Procedure/treatment should be performed/administered  **Class IIA** = It is reasonable to perform procedure/administer treatment  **Class IIB** = Procedure/Treatment may be considered  **Class III No Benefit** /**Class III Harm** | | Class III No Benefit; Class III Harm | |
| ESC  (2019, 2017, 2013) | **Class I** = Is recommended or is indicated **Class II** = Conflicting evidence and/or a divergence of opinion about the usefulness/efficacy of the given treatment or procedure  **Class IIa** = Should be considered  **Class IIb** = May be considered  **Class III** = Is not recommended | | Class III | |
| *NSCLC* | | | | |
| ASCO  (2004, 2009, 2011) | N/A | | N/A | |
| ASCO  (2015, 2017, 2020, 2021) | **Strong**: There is high confidence that the recommendation reflects best practice. This is based on: a) strong evidence for a true net effect (e.g. benefits exceed harms); b) consistent results, with no or minor exceptions; c) minor or no concerns about study quality; and/or d) the extent of panelists’ agreement. Other compelling considerations (discussed in the guideline’s literature review and analyses) may also warrant a strong recommendation  **Moderate**: There is moderate confidence that the recommendation reflects best practice. This is based on: a) good evidence for a true net effect (e.g. benefits exceed harms); b) consistent results, with minor and/or few exceptions; c) minor and/or few concerns about study quality; and/or d) the extent of panelists’ agreement. Other compelling considerations (discussed in the guideline’s literature review and analyses) may also warrant a moderate recommendation **Weak:** There is some confidence that the recommendation offers the best current guidance for practice. This is based on: a) limited evidence for a true net effect (e.g. benefits exceed harms); b) consistent results, but with important exceptions; c) concerns about study quality; and/or d) the extent of panelists’ agreement. Other considerations (discussed in the guideline’s literature review and analyses) may also warrant a weak recommendation | | Weak | |
| ESMO  (All years) | **A** = Strong evidence for efficacy with a substantial clinical benefit, strongly recommended **B** = Strong or moderate evidence for efficacy but with a limited clinical benefit, generally recommended **C** = Insufficient evidence for efficacy or benefit does not outweigh the risk or the disadvantages (adverse events, costs), optional **D** = Moderate evidence against efficacy or for adverse outcome, generally not recommended **E =** Strong evidence against efficacy or for adverse outcome, never recommended | | C; D; E | |
| *COPD* | | | | |
| GOLD  (All years) | | N/A (Only had evidence rating)** | N/A | |
| VA DoD  (2021 & 2014) | | **Strong for** = We recommend **Weak for** = We suggest **Neither for nor against** = There is insufficient evidence to recommend for or against **Weak against** = We suggest against **Strong against** = We recommend against | Neither for nor against; Weak against; Strong against | |
| VA DoD  (2007) | | **A** = A strong recommendation that the clinicians provide the intervention to eligible patients **B** = A recommendation that clinicians provide (the service) to eligible patients **C** = No recommendation for or against the routine provision of the intervention is made **D** = Recommendation is made against routinely providing the intervention to asymptomatic patients **I** = The conclusion is that the evidence is insufficient to recommend for or against routinely providing the intervention | C; D; I | |
| ERS/ATS  (2017) | | **Strong** = Made for an intervention when the panel was certain that the desirable consequences of the intervention outweighed the undesirable consequences, just as a strong recommendation would have been made against an intervention if the panel was certain that the undesirable consequences of the intervention outweigh the desirable consequences **Conditional** = Made for an intervention when the panel was uncertain that the desirable consequences of the intervention outweighed the undesirable consequences, just as a conditional recommendation would have been made against an intervention if the panel was uncertain that the undesirable consequences of the intervention outweigh the desirable consequences | None | |
| AAFP  (2010) | | **A** = Consistent, good-quality patient-oriented evidence **B** = Inconsistent or limited-quality patient-oriented evidence **C** = Consensus, disease-oriented evidence, usual practice, expert opinion, or case series | C | |
| *AD* | | | | |
| APA  (2014) | | N/A | | N/A |
| APA  (2007) | | **I.)** Recommended with substantial clinical confidence **II.)** Recommended with moderate clinical confidence **III.)** May be recommended on the basis of individual circumstances | | III.) |
| AAFP/ACP  (2008) | | **Strong** = Benefits clearly outweigh risks and burden OR risks and burden clearly outweigh benefits **Weak** = Benefits finely balanced with risks and burden | | Weak |
| AAFP  (2002) | | N/A | | N/A |
| EFNS/EAN  (2007 & 2010) | | **A** = Established as effective, ineffective, or harmful **B** = Probably effective, ineffective, or harmful **C** = Possibly effective, ineffective, or harmful | | C |
| EFNS/EAN  (2015) | | **Strong** = When the desirable effects of an intervention clearly outweigh the undesirable effects, or clearly do not **Weak** = When the trade-offs are less certain—either because of low quality evidence or because evidence suggests that desirable and undesirable effects are closely balanced | | Weak |
| *T2D* | | | | |
| AACE/ACE  (2015 to 2020) | | Length of line in treatment algorithm diagram reflects strength of recommendation | | N/A*** |
| AACE/ACE (2013,2009) | | N/A | | N/A |
| AACE/ACE  (2007) | | **Grade A** = Homogeneous evidence from multiple well-designed randomized controlled trials with sufficient statistical power; Homogeneous evidence from multiple well-designed cohort controlled trials with sufficient statistical power; ≥1 conclusive level-of-evidence category 1 publications demonstrating benefit>>risk **Grade B** = Evidence from at least one large well-designed clinical trial, cohort or case-controlled analytic study, or meta-analysis; No conclusive level-of-evidence category 1 publication; ≥1 conclusive level-of evidence category 2 publications demonstrating benefit>>risk **Grade C** = Evidence based on clinical experience, descriptive studies, or expert consensus opinion; No conclusive level-of-evidence category 1 or 2 publication; ≥1 conclusive level of-evidence category 3 publications demonstrating benefit>>risk; No conclusive risk at all and no conclusive benefit demonstrated by evidence **Grade D** = Not rated; No conclusive level-of-evidence category 1, 2, or 3 publication demonstrating benefit>>risk; Conclusive level-of-evidence category 1, 2, or 3 publication demonstrating risk>>benefit  (An A grade is the strongest recommendation, and a D grade is the weakest recommendation) | | C; D |
| ADA  (All years) | | N/A *(Only had evidence rating)*** | | N/A |
| EASD/ADA  (All years) | | N/A | | N/A |
| IDF  (All years) | | N/A | | N/A |
| ^If the guideline did not provide a strength of recommendation, statements in the guidelines were used to discern whether the guideline was recommending a therapy  *US guidelines were published by multiple organizations including ACCF/AHA/ACP/AATS/PCNA/SCAI/STS (2012); ACC/AHA/AATS/PCNA/SCAI/STS (2014); ACC/AHA (2016)  **Guidelines only provided grading for levels of evidence  ***All were included as no specific length was defined as 'weak'  IHD = Ischemic heart disease; NSCLC = Non-small cell lung cancer; COPD = Chronic obstructive pulmonary disease; AD = Alzheimer’s disease; T2D = Type 2 diabetes; US = United States  Organization abbreviations:  AACE = American Association of Clinical Endocrinology  AAFP = American Academy of Family Physicians  AATS = American Association for Thoracic Surgery  ACC = American College of Cardiology  ACCF = American College of Cardiology Foundation  ACE = American College of Endocrinology  ACP = American College of Physicians  ADA = American Diabetes Association  AHA = American Heart Association  APA = American Psychiatric Association  ASCO = American Society of Clinical Oncology  ATS = American Thoracic Society  DoD = Department of Defense  EASD = European Association for the Study of Diabetes  EAN = European Academy of Neurology  EFNS = European Federation of Neurological Sciences  ERS = European Respiratory Society  ESC = European Society of Cardiology  ESMO = European Society for Medical Oncology  GOLD = Global Initiative for Chronic Obstructive Lung Disease  IDF = International Diabetes Federation  OH = Ontario Health  PCNA = Preventive Cardiovascular Nurses Association  SCAI = Society for Cardiovascular Angiography and Interventions  STS = Society of Thoracic Surgeons  VA = Department of Veteran Affairs | | | | |

**Supplementary Table S2. Guidelines Abstracted by Disease Area**

| Region | Year | Organization(s)* | Title |
| --- | --- | --- | --- |
| **IHD** | | | |
| US | 2012 | ACCF/AHA/ACP/AATS/PCNA/SCAI/ STS | 2012 ACCF/AHA/ACP/AATS/PCNA/SCAI/ STS Guideline for the Diagnosis and Management of Patients with Stable Ischemic Heart Disease |
| US | 2014 | ACC/AHA/AATS/PCNA/SCAI/STS | 2014 ACC/AHA/AATS/PCNA/SCAI/STS Focused Update of the Guideline for the Diagnosis and Management of Patients with Stable Ischemic Heart Disease |
| US | 2016 | ACC/AHA | 2016 ACC/AHA Guideline Focused Update on Duration of Dual Antiplatelet Therapy in Patients with Coronary Artery Disease: A Report of the American College of Cardiology/American Heart Association Task Force on Clinical Practice Guidelines |
| EU | 2013 | ESC | 2013 ESC guidelines on the management of stable coronary artery disease |
| EU | 2017 | ESC | 2017 ESC focused update on dual antiplatelet therapy in coronary artery disease developed in collaboration with EACTS: The Task Force for dual antiplatelet therapy in coronary artery disease of the European Society of Cardiology (ESC) and of the European Association for Cardio-Thoracic Surgery (EACTS) |
| EU | 2019 | ESC | 2019 ESC Guidelines for the diagnosis and management of chronic coronary syndromes: The Task Force for the diagnosis and management of chronic coronary syndromes of the European Society of Cardiology (ESC) |
| **NSCLC** | | | |
| US | 2004 | ASCO | American Society of Clinical Oncology Treatment of Unresectable Non–Small-Cell Lung Cancer Guideline: Update 2003 |
| US | 2009 | ASCO | American Society of Clinical Oncology Clinical Practice Guideline Update on Chemotherapy for Stage IV Non–Small-Cell Lung Cancer |
| US | 2011 | ASCO | 2011 Focused Update of 2009 American Society of Clinical Oncology Clinical Practice Guideline Update on Chemotherapy for Stage IV Non–Small-Cell Lung Cancer |
| US | 2015 | ASCO | Systemic Therapy for Stage IV Non–Small-Cell Lung Cancer: American Society of Clinical Oncology Clinical Practice Guideline Update |
| US | 2017 | ASCO | Systemic Therapy for Stage IV Non–Small-Cell Lung Cancer: American Society of Clinical Oncology Clinical Practice Guideline Update |
| US | 2020 | ASCO/OH | Therapy for Stage IV Non–Small-Cell Lung Cancer Without Driver Alterations: ASCO and OH (CCO) Joint Guideline Update |
| US | 2021 | ASCO/OH | Therapy for Stage IV Non–Small-Cell Lung Cancer with Driver Alterations: ASCO and OH (CCO) Joint Guideline Update |
| EU | 2001 | ESMO | ESMO Minimum Clinical Recommendations for diagnosis, treatment and follow-up of non-small-cell lung cancer (NSCLC) |
| EU | 2005 | ESMO | ESMO Minimum Clinical Recommendations for diagnosis, treatment and follow-up of non-small-cell lung cancer (NSCLC) |
| EU | 2007 | ESMO | Non-small-cell lung cancer: ESMO clinical recommendations for diagnosis, treatment and follow-up |
| EU | 2008 | ESMO | Non-small-cell lung cancer: ESMO clinical recommendations for diagnosis, treatment and follow-up |
| EU | 2009 | ESMO | Non-small-cell lung cancer: ESMO clinical recommendations for diagnosis, treatment and follow-up |
| EU | 2010 | ESMO | Metastatic non-small-cell lung cancer: ESMO Clinical Practice Guidelines for diagnosis, treatment and follow-up |
| EU | 2012 | ESMO | Metastatic non-small-cell lung cancer (NSCLC): ESMO Clinical Practice Guidelines for diagnosis, treatment and follow-up |
| EU | 2014 | ESMO | Metastatic non-small-cell lung cancer (NSCLC): ESMO Clinical Practice Guidelines for diagnosis, treatment and follow-up |
| EU | 2016 | ESMO | Metastatic non-small-cell lung cancer: ESMO Clinical Practice Guidelines for diagnosis, treatment and follow-up |
| EU | 2018 | ESMO | Metastatic non-small cell lung cancer: ESMO Clinical Practice Guidelines for diagnosis, treatment and follow-up |
| EU | 2019 | ESMO | Metastatic non-small cell lung cancer: ESMO Clinical Practice Guidelines for diagnosis, treatment and follow-up |
| EU | 2020 | ESMO | Metastatic non-small cell lung cancer: ESMO Clinical Practice Guidelines for diagnosis, treatment and follow-up (Updated Version) |
| **COPD** | | | |
| Global | 2001 | GOLD | Global Strategy for the Diagnosis, Management, and Prevention of Chronic Obstructive Pulmonary Disease NHLBI/WHO Global Initiative for Chronic Obstructive Lung Disease (GOLD) Workshop Summary |
| Global | 2003 | GOLD | Global Strategy for the Diagnosis, Management and Prevention of COPD: 2003 update |
| Global | 2004 | GOLD | Global Strategy for the Diagnosis, Management and Prevention of COPD: Updated 2004 |
| Global | 2005 | GOLD | Global Strategy for the Diagnosis, Management and Prevention of COPD: Updated 2005 |
| Global | 2006 | GOLD | Global Strategy for the Diagnosis, Management and Prevention of COPD (2006) |
| Global | 2007 | GOLD | Global Strategy for the Diagnosis, Management, and Prevention of Chronic Obstructive Pulmonary Disease GOLD Executive Summary |
| Global | 2008 | GOLD | Global Strategy for the Diagnosis, Management, and Prevention of Chronic Obstructive Pulmonary Disease (Updated 2008) |
| Global | 2009 | GOLD | Global Strategy for the Diagnosis, Management, and Prevention of Chronic Obstructive Pulmonary Disease (Updated 2009) |
| Global | 2010 | GOLD | Global Strategy for the Diagnosis, Management, and Prevention of Chronic Obstructive Pulmonary Disease (Updated 2010) |
| Global | 2011 | GOLD | Global Strategy for the Diagnosis, Management, and Prevention of Chronic Obstructive Pulmonary Disease (Revised 2011) |
| Global | 2013 | GOLD | Global Strategy for the Diagnosis, Management, and Prevention of Chronic Obstructive Pulmonary Disease GOLD Executive Summary |
| Global | 2014 | GOLD | Global Strategy for the Diagnosis, Management, and Prevention of Chronic Obstructive Pulmonary Disease Updated 2014 |
| Global | 2015 | GOLD | Global Strategy for the Diagnosis, Management, and Prevention of Chronic Obstructive Pulmonary Disease Updated 2015 |
| Global | 2016 | GOLD | Global Strategy for the Diagnosis, Management, and Prevention of Chronic Obstructive Pulmonary Disease Updated 2016 |
| Global | 2017 | GOLD | Global Strategy for the Diagnosis, Management, and Prevention of Chronic Obstructive Pulmonary Disease 2017 Report |
| Global | 2018 | GOLD | Global Strategy for the Diagnosis, Management, and Prevention of Chronic Obstructive Pulmonary Disease 2018 Report |
| Global | 2019 | GOLD | Global Strategy for the Diagnosis, Management, and Prevention of Chronic Obstructive Pulmonary Disease 2019 Report |
| Global | 2020 | GOLD | Global Strategy for the Diagnosis, Management, and Prevention of Chronic Obstructive Pulmonary Disease 2020 Report |
| Global | 2021 | GOLD | Global Strategy for the Diagnosis, Management, and Prevention of Chronic Obstructive Pulmonary Disease 2021 Report |
| US | 2007 | VA/DoD | VA/DoD Clinical Practice Guideline for Management of Outpatient Chronic Obstructive Pulmonary Disease |
| US | 2010 | AAFP | Management of COPD Exacerbations |
| US | 2014 | VA/DoD | VA/DoD Clinical Practice Guideline for the Management of Chronic Obstructive Pulmonary Disease |
| US | 2021 | VA/DoD | VA/DoD Clinical Practice Guideline for the Management of Chronic Obstructive Pulmonary Disease |
| US/EU | 2017 | ERS/ATS | Management of COPD Exacerbations: A European Respiratory Society/American Thoracic Society Guideline |
| **AD** | | | |
| US | 2002 | AAFP | Guidelines for managing Alzheimer's disease: Part II. Treatment |
| US | 2007 | APA | American Psychiatric Association practice guideline for the treatment of patients with Alzheimer's disease and other dementias. Second edition |
| US | 2008 | AAFP/ACP | Current pharmacologic treatment of dementia: a clinical practice guideline from the American College of Physicians and the American Academy of Family Physicians |
| US | 2014 | APA | Guideline Watch (October 2014): Practice Guideline for the Treatment of Patients with Alzheimer's Disease and Other Dementias |
| EU | 2007 | EFNS/EAN | Recommendations for the diagnosis and management of Alzheimer's disease and other disorders associated with dementia: EFNS guideline |
| EU | 2010 | EFNS/EAN | EFNS guidelines for the diagnosis and management of Alzheimer’s disease |
| EU | 2015 | EFNS/EAN | EFNS-ENS/EAN Guideline on concomitant use of cholinesterase inhibitors and memantine in moderate to severe Alzheimer's disease |
| **T2D** | | | |
| US | 2002 | ADA | Standards of Medical Care for Patients With Diabetes Mellitus |
| US | 2003 | ADA | Standards of Medical Care for Patients with Diabetes Mellitus |
| US | 2004 | ADA | Standards of Medical Care in Diabetes |
| US | 2005 | ADA | Standards of Medical Care in Diabetes |
| US | 2006 | ADA | Standards of Medical Care in Diabetes—2006 |
| US | 2007 | ADA | Standards of Medical Care in Diabetes—2007 |
| US | 2008 | ADA | Standards of Medical Care in Diabetes—2008 |
| US | 2009 | ADA | Standards of Medical Care in Diabetes—2009 |
| US | 2010 | ADA | Standards of Medical Care in Diabetes—2010 |
| US | 2011 | ADA | Standards of Medical Care in Diabetes—2011 |
| US | 2012 | ADA | Standards of Medical Care in Diabetes—2012 |
| US | 2013 | ADA | Standards of Medical Care in Diabetes—2013 |
| US | 2014 | ADA | Standards of Medical Care in Diabetes—2014 |
| US | 2015 | ADA | Standards of Medical Care in Diabetes—2015 |
| US | 2016 | ADA | Standards of Medical Care in Diabetes—2016 |
| US | 2017 | ADA | Standards of Medical Care in Diabetes—2017 |
| US | 2018 | ADA | Standards of Medical Care in Diabetes-—2018 |
| US | 2019 | ADA | Standards of Medical Care in Diabetes— 2019 |
| US | 2020 | ADA | Standards of Medical Care in Diabetes— 2020 |
| US | 2021 | ADA | Standards of Medical Care in Diabetes— 2021 |
| US | 2007 | AACE | American Association of Clinical Endocrinologists Medical Guidelines for Clinical Practice for the Management of Diabetes Mellitus |
| US | 2009 | AACE/ACE | Statement by an American Association of Clinical Endocrinologists/ American College of Endocrinology Consensus Panel on Type 2 Diabetes Mellitus: An Algorithm for Glycemic Control |
| US | 2013 | AACE | American Association of Clinical Endocrinologists Comprehensive Diabetes Management Algorithm 2013 Consensus Statement |
| US | 2015 | AACE/ACE | Consensus Statement by the American Association of Clinical Endocrinologists and American College of Endocrinology on the Comprehensive Type 2 Diabetes Management Algorithm - 2015 Executive Summary |
| US | 2016 | AACE/ACE | Consensus Statement by The American Association of Clinical Endocrinologists and American College of Endocrinology on The Comprehensive Type 2 Diabetes Management Algorithm – 2016 Executive Summary |
| US | 2017 | AACE/ACE | Consensus Statement by the American Association of Clinical Endocrinologists and American College of Endocrinology on the Comprehensive type 2 Diabetes Management Algorithm – 2017 Executive Summary |
| US | 2018 | AACE/ACE | Consensus Statement by the American Association of Clinical Endocrinologists and American College of Endocrinology on the Comprehensive Type 2 Diabetes Management Algorithm – 2018 Executive Summary |
| US | 2019 | AACE/ACE | Consensus Statement by the American Association of Clinical Endocrinologists and American College of Endocrinology on the Comprehensive Type 2 Diabetes Management Algorithm – 2019 Executive Summary |
| US | 2020 | AACE/ACE | Consensus Statement by the American Association of Clinical Endocrinologists and American College of Endocrinology on the Comprehensive Type 2 Diabetes Management Algorithm – 2020 Executive Summary |
| EU-US joint | 2006 | ADA/EASD | Management of Hyperglycemia in Type 2 Diabetes: A Consensus Algorithm for the Initiation and Adjustment of Therapy A consensus statement from the American Diabetes Association and the European Association for the Study of Diabetes |
| EU-US joint | 2009 | ADA/EASD | Medical Management of Hyperglycemia in Type 2 Diabetes: A Consensus Algorithm for the Initiation and Adjustment of Therapy A consensus statement of the American Diabetes Association and the European Association for the Study of Diabetes |
| EU-US joint | 2012 | ADA/EASD | Management of Hyperglycemia in Type 2 Diabetes: A Patient-Centered Approach Position Statement of the American Diabetes Association (ADA) and the European Association for the Study of Diabetes (EASD) |
| EU-US joint | 2015 | ADA/EASD | Management of hyperglycaemia in type 2 diabetes, 2015: a patient-centred approach. Update to a Position Statement of the American Diabetes Association and the European Association for the Study of Diabetes |
| EU-US joint | 2018 | ADA/EASD | Management of hyperglycaemia in type 2 diabetes, 2018. A consensus report by the American Diabetes Association (ADA) and the European Association for the Study of Diabetes (EASD) |
| Global | 2005 | IDF | Global Guideline for Type 2 Diabetes |
| Global | 2012 | IDF | Global Guideline for Type 2 Diabetes |

| IHD = Ischemic heart disease; NSCLC = Non-small cell lung cancer; COPD = Chronic obstructive pulmonary disease; AD = Alzheimer’s disease; T2D = Type 2 diabetes; US = United States; EU = European Union  *Organization abbreviations:  AACE = American Association of Clinical Endocrinology  AAFP = American Academy of Family Physicians  AATS = American Association for Thoracic Surgery  ACC = American College of Cardiology  ACCF = American College of Cardiology Foundation  ACE = American College of Endocrinology  ACP = American College of Physicians  ADA = American Diabetes Association  AHA = American Heart Association  APA = American Psychiatric Association  ASCO = American Society of Clinical Oncology  ATS = American Thoracic Society  DoD = Department of Defense  EASD = European Association for the Study of Diabetes  EAN = European Academy of Neurology  EFNS = European Federation of Neurological Sciences  ERS = European Respiratory Society  ESC = European Society of Cardiology  ESMO = European Society for Medical Oncology  GOLD = Global Initiative for Chronic Obstructive Lung Disease  IDF = International Diabetes Federation  OH = Ontario Health  PCNA = Preventive Cardiovascular Nurses Association  SCAI = Society for Cardiovascular Angiography and Interventions  STS = Society of Thoracic Surgeons  VA = Department of Veteran Affairs |
| --- |

**Supplementary Figure S3. Pace of Pharmacotherapy Adoption in IHD A) US and B) EU Guidelines**

1. **US Guidelines**


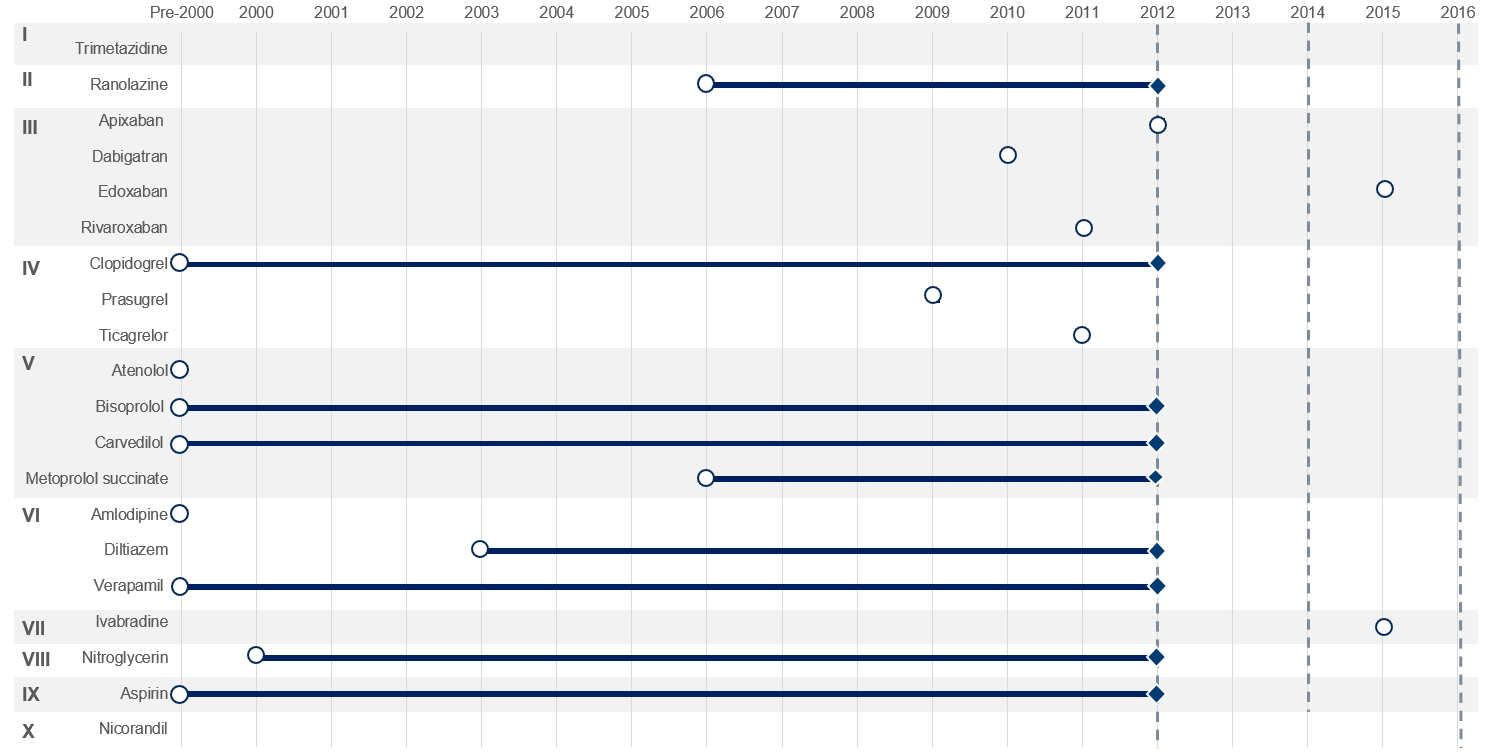


1. **EU Guidelines**


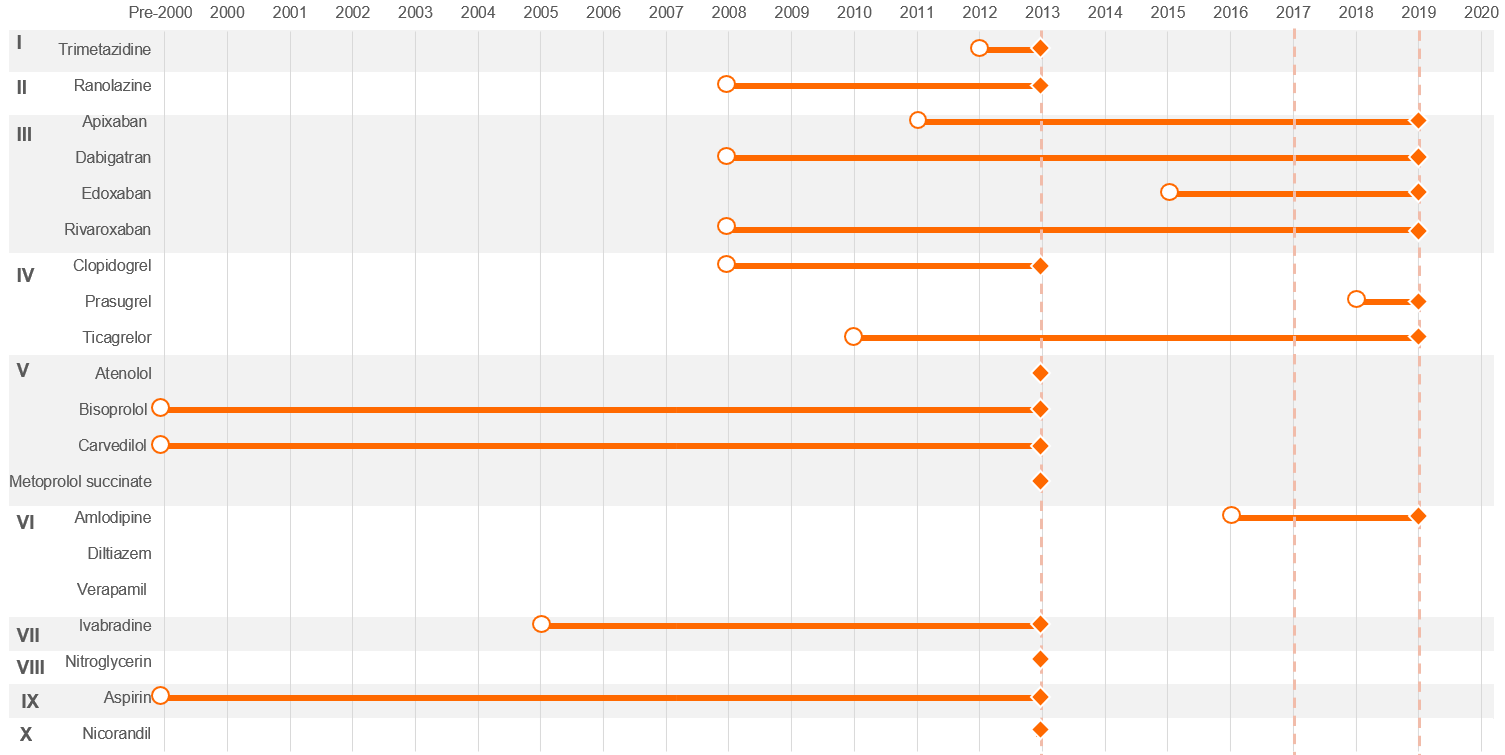


Classes of pharmacotherapies: I.) 3-KAT inhibitors; II.) Anti-anginal; III.) Anticoagulants; IV.) Antiplatelet therapy; V.) Beta-blocker therapy; VI.) Calcium channel blocker; VII.) HCN channel blocker; VIII.) Nitrates; IX.) NSAID; X.) Potassium channel activator

Note: Atenolol, Metoprolol succinate, Diltiazem, Verapamil, Nitroglycerin, and Nicorandil are approved in select EU countries but have not been formally reviewed and approved by the EMA. Bisoprolol and Aspirin were approved prior to the establishment of the EMA.

IHD = Ischemic heart disease; US = United States; EU = European Union; EMA = European Medicines Agency

Figure S3A Legend:

Guideline published

Year approved by FDA

Year first recommended in guideline

Figure S3B Legend:

Guideline published

Year approved by EMA

Year first recommended in guideline

**Supplementary Figure S4. Pace of Pharmacotherapy Adoption in COPD A) US Guidelines**

1. **US Guidelines**


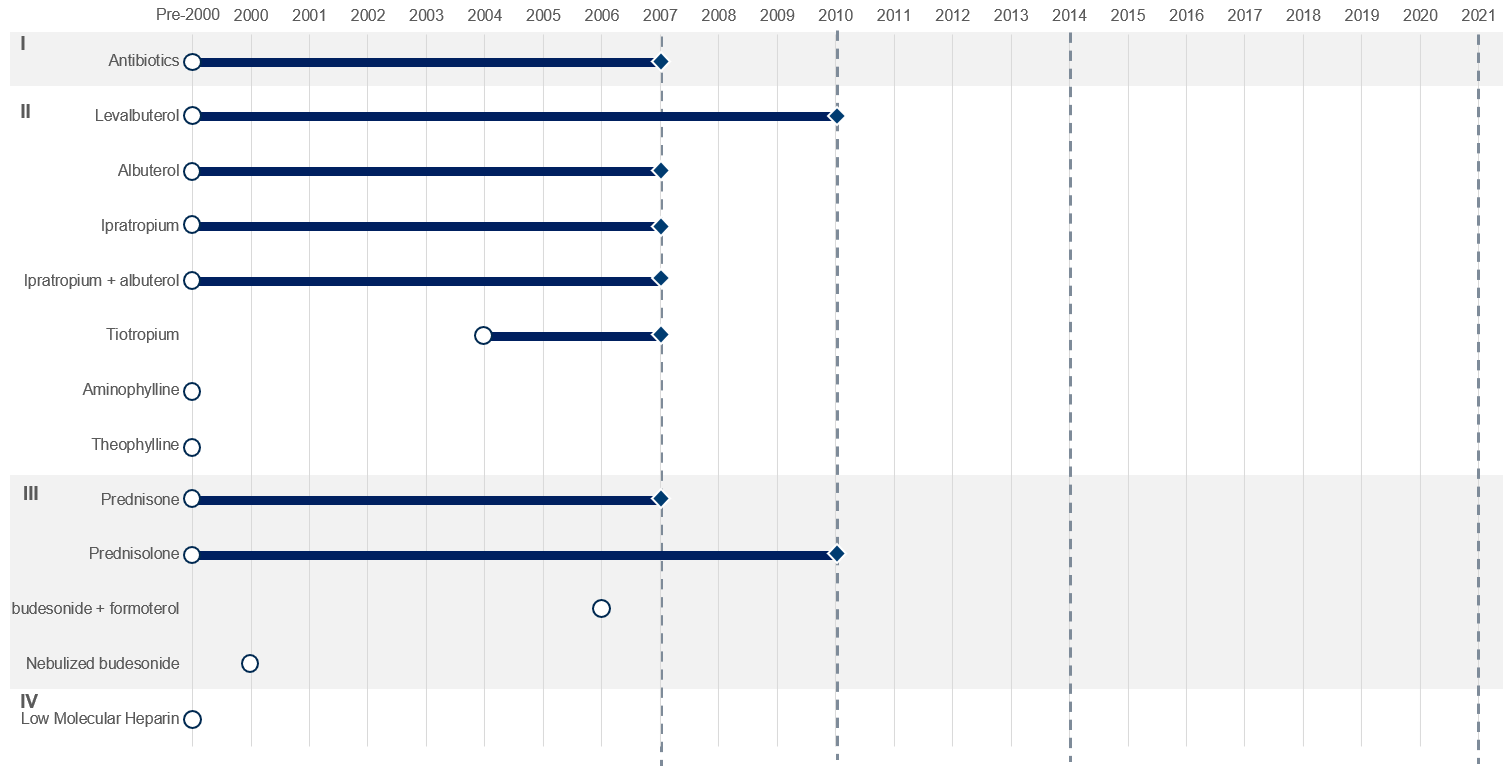


Classes of pharmacotherapies: I.) Antibiotics; II.) Bronchodilator therapy; III.) Glucocorticosteroids; IV.) Anticoagulant

Note: There were no EU specific guidelines for COPD identified so this analysis was only done for the US guidelines

COPD = Chronic obstructive pulmonary disease; US = United States

Figure S4A Legend:

Guideline published

Year approved by FDA

Year first recommended in guideline

**Supplementary Figure S5. Pace of Pharmacotherapy Adoption in AD A) US and B) EU guidelines**

1. **US Guidelines**


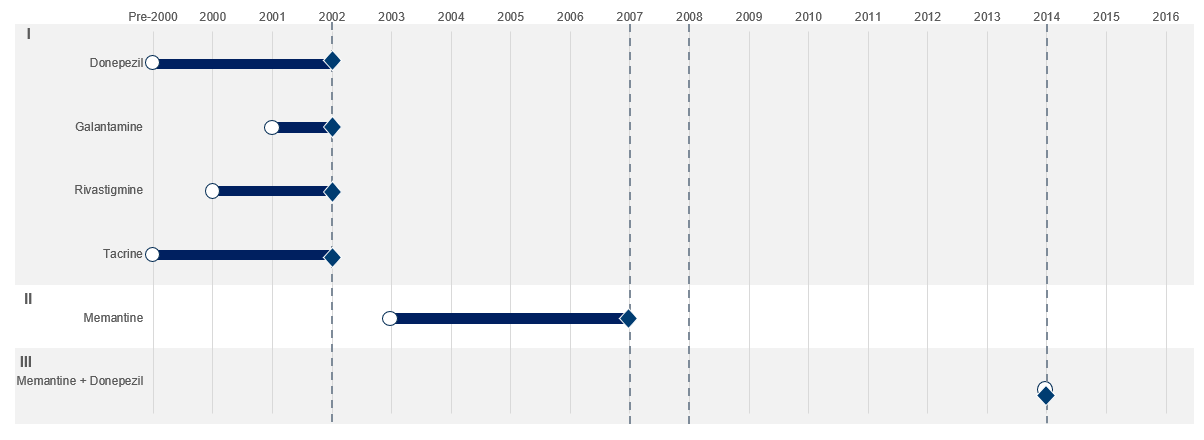


1. **EU Guidelines**


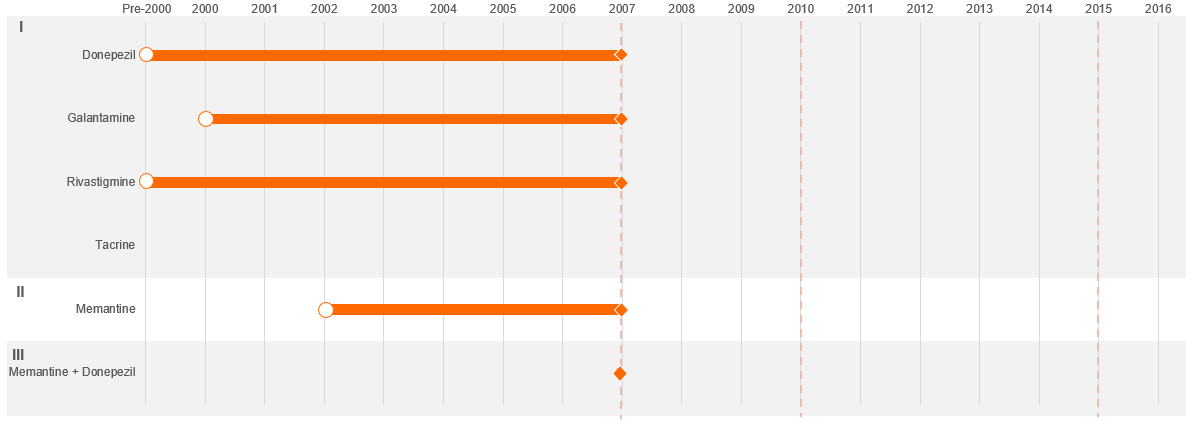


Classes of pharmacotherapies: I.) Cholinesterase Inhibitors; II.) Neuropeptide Modifying Agent; III.) Combination Neuropeptide Modifying Agent + Cholinesterase Inhibitor

Note: Tacrine was approved in select EU countries but has not been formally reviewed and approved by the EMA

AD = Alzheimer’s disease; US = United States; EU = European Union; EMA = European Medicines Agency

Figure S5A Legend:

Guideline published

Year approved by FDA

Year first recommended in guideline

Figure S5B Legend:

Guideline published

Year approved by EMA

Year first recommended in guideline

**Supplementary Figure S6. Pace of Pharmacotherapy Adoption in T2D A) US (ADA), B) US (AACE/ACE), and C) EU Guidelines**

1. **US (ADA) Guidelines**


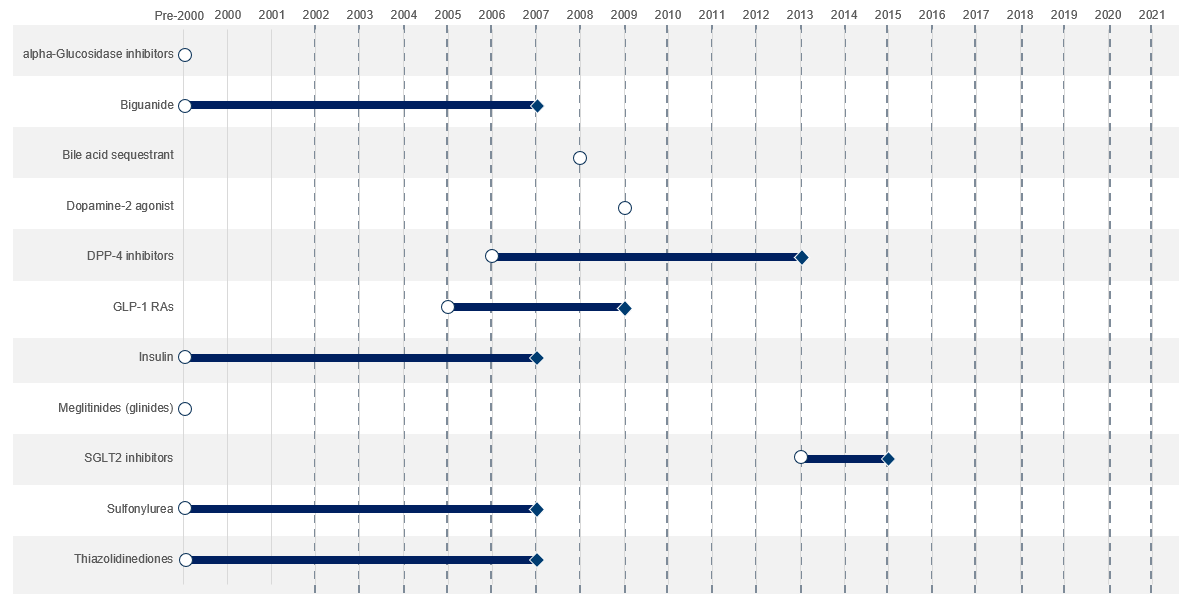


1. **US (AACE/ACE) Guidelines**


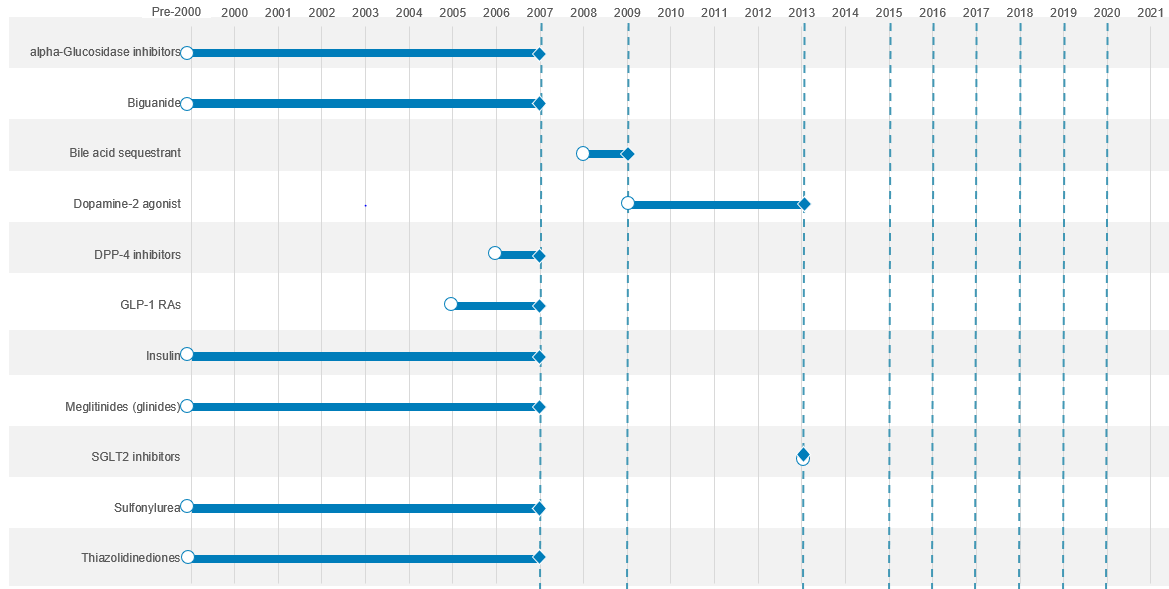


1. **EU Guidelines**


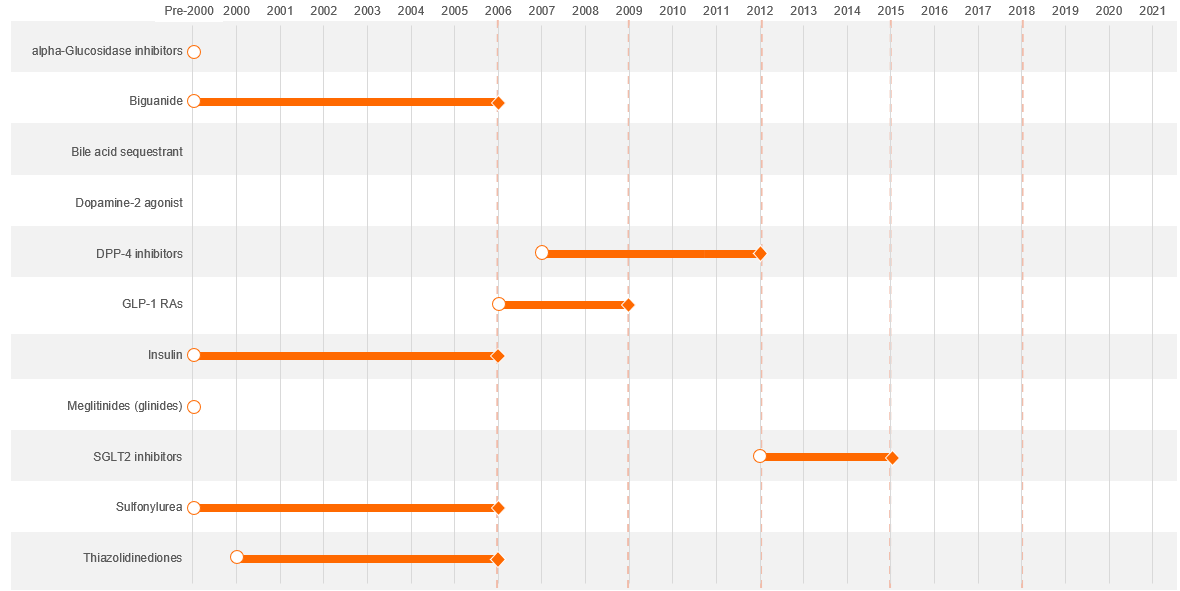


Note: T2D guidelines made recommendations at the drug class level so year of approval was based on the year the first drug in the class was approved. Alpha-Glucosidase inhibitors, Metformin, Insulin, Meglitinides, and Sulfonylureas were approved prior to establishment of the EMA.

AACE = American Association of Clinical Endocrinology; ACE = American College of Endocrinology; ADA = American Diabetes AssociationT2D = Type 2 diabetes; US = United States; EU = European Union; EMA = European Medicines Agency

Figure S6A Legend:

Guideline published

Year approved by FDA

Year first recommended in guideline

Figure S6B Legend:

Guideline published

Year approved by FDA

Year first recommended in guideline

Figure S6C Legend:

Guideline published

Year approved by EMA

Year first recommended in guideline
